# The Prevalence of Cognitive Impairment on Admission to Nursing Home among Residents with and without Stroke: A Cross–Sectional Survey of Nursing Homes in Ireland

**DOI:** 10.3390/ijerph17197203

**Published:** 2020-10-01

**Authors:** Nora-Ann Donnelly, Eithne Sexton, Niamh A. Merriman, Kathleen E. Bennett, David J Williams, Frances Horgan, Paddy Gillespie, Anne Hickey, Maev-Ann Wren

**Affiliations:** 1 Division of Population Health Sciences, Royal College of Surgeons in Ireland, D02 P796, Ireland; eithnesexton@rcsi.ie (E.S.); niamhmerriman@rcsi.ie (N.A.M.); kathleenebennett@rcsi.ie (K.E.B.); ahickey@rcsi.ie (A.H.); 2 Social Research Division, Economic and Social Research Institute, D02 K138, Ireland; maev-ann.wren@esri.ie; 3 Department of Geriatric and Stroke Medicine, Royal College of Surgeons in Ireland, D02 P796, Ireland; davidwilliams@rcsi.ie; 4 Department Physiotherapy, Royal College of Surgeons in Ireland, D02 P796, Ireland; fhorgan@rcsi.ie; 5 Health Economics & Policy Analysis Centre (HEPAC), Department of Economics, NUI Galway, H91 TK33, Ireland; paddy.gillespie@nuigalway.ie

**Keywords:** stroke, cognitive impairment, nursing home residents

## Abstract

Post–stroke cognitive impairment (PSCI) is a common consequence of stroke. Epidemiological evidence indicates that, with an ageing population, stroke and PSCI are likely to increase in the coming decades. This may have considerable implications for the demand for nursing home placement. As prevalence estimates of both cognitive impairment and dementia on admission to nursing home among residents with and without stroke have not yet been compared, they were estimated and compared in this study. We performed a cross–sectional survey to establish the admission characteristics of 643 residents in 13 randomly selected nursing homes in Ireland. The survey collected data on resident’s stroke and cognitive status at the time of nursing home admission. The survey found, among nursing home residents that experienced stroke prior to admission, prevalence estimates for cognitive impairment (83.8%; 95% CI = 76.9–90.6%) and dementia (66.7%; 95% CI = 57.9–75.4%) were significantly higher compared to residents that had not experienced stroke prior to admission (cognitive impairment: 56.6%; 95% CI = 52.4–60.8%; X^2^ (1) = 28.64; *p* < 0.001; dementia: 49.8%; 95% CI = 45.6–54.1%; X^2^ (1) = 10.47; *p* < 0.01). Since the prevalence of PSCI is likely to increase in the coming decades, the findings highlight an urgent need for health service planning for this increased demand for nursing home care to meet the care needs of these stroke survivors.

## 1. Introduction

Stroke is a principal cause of morbidity and mortality internationally [1,2]. In Ireland, it is estimated that the highest proportion of the national costs of stroke arise out of nursing home care (NHC), at almost 50% of total stroke costs [3]. Stroke has been found to be a strong, independent and potentially modifiable risk factor for cognitive impairment and dementia [4]. It is estimated that, in the first year following stroke, 26.5% of stroke survivors have dementia, and 38% of survivors experience cognitive impairment that does not meet the criteria for dementia (cognitive impairment no dementia (CIND)) [5,6]. Yet post–stroke cognitive impairment (PSCI) is an often overlooked consequence of stroke [5,6,7,8]. While there is a considerable body of knowledge surrounding interventions to improve physical function post–stroke, there is significantly less research dedicated to cognitive impairment [7,9]. Indeed, understanding and reducing cognitive impairment post–stroke is regarded as “the single most important topic in stroke research” [10,11,12].

It is widely recognised that cognitive impairment is a major predictor of transition to nursing home [13]. Studies that have examined the risk of nursing home entry for stroke survivors found those experiencing PSCI to have an estimated three– to four–fold increased risk of nursing home placement following stroke [14,15]. While the risk of nursing home entry for those with PSCI has been examined, results have varied by study setting, sample size and follow–up periods. Consequently, the strength of the effect of PSCI on nursing home placement has varied between studies [16]. The characteristics of stroke survivors admitted to nursing homes have been examined in a number of studies [17,18,19,20,21]. These studies found high levels of care needs amongst stroke survivors residing in nursing homes. However, this research did not examine the prevalence of both cognitive impairment and dementia amongst stroke survivors. The extent of the utilisation of nursing home care both for those with cognitive impairment and for those with dementia post–stroke has yet to be determined. These data are important for health service planners and policy makers, as epidemiological evidence suggests that, as the risks of stroke and cognitive impairment increase exponentially with age, PSCI is likely to become more prevalent in the coming decades, in line with an ageing population [22,23,24]. This is likely to have considerable implications for the demand for nursing home care places to meet this growing demand. Therefore, this study aimed to estimate the prevalence of both cognitive impairment and dementia on admission to nursing home for residents with and without stroke in Ireland.

## 2. Materials and Methods

### 2.1. Study Design, Setting and Population

This study has been reported in accordance with the STROBE checklist [25]. This was a cross–sectional survey of nursing homes in Ireland in 2018. As all facilities providing long–stay residential beds for older people are required to register with the Health Information and Quality Authority (HIQA) [26], the HIQA register of nursing home beds formed the sample frame for this study. According to this register, the average size of a nursing home in Ireland is approximately 52 beds. As there is no annual census of nursing home residents in Ireland, the choice of year for the sampling frame was dictated by data availability. The average occupancy rate for long–stay residential facilities nationally was estimated to be 94% in 2015 [27]. Therefore, the total number of residents in HIQA registered nursing homes was estimated to be 28,300.

### 2.2. Power Calculation and Sample Size

To determine the number of residents with cognitive impairment or dementia on admission that have or have not experienced stroke prior to admission to the nursing home, a random sample of 643 residents was selected. The sample size was sufficiently large to be able to provide a 95% confidence interval within +/− 5% precision on any estimate [28]. The sample size was derived by applying the Cochran formula to derive the sample size for categorical variables [28,29]. The required minimum returned sample was 379 residents. The anticipated response rate was 56% and the final response rate was 59%, bringing the final returned sample up to 643 residents. These residents came from 13 randomly selected nursing homes nationally, with an average of 49 residents in each nursing home. It is estimated that, in Ireland, 77% of nursing homes are operated by private providers and 23% by public providers [26]. Consequently, the number of randomly selected nursing homes and nursing home residents in each region of the country was stratified to reflect the proportion of the nursing home population and nursing home ownership (public/private) within that region.

### 2.3. Data Collection and Measures

The survey questionnaire was developed for this study (see supplementary material Additional File 1 Survey Questionnaire). The survey questionnaire was completed via face–to–face interviews with Directors of Nursing (DON) in each nursing home. The questionnaire was posted to each nursing home in advance of the interview. DONs examined the charts of each resident to check their stroke and cognitive status at the time of nursing home placement, that is, whether the resident had experienced stroke prior to admission and their cognitive status on admission. The extracted data were aggregated and completed on the survey questionnaire during face–to–face interviews.

The survey data was collected in this manner for a number of reasons. Firstly, as the survey was conducted to estimate the prevalence of both cognitive impairment and dementia on admission to nursing home among residents with and without stroke, the questionnaire collected aggregated data on resident’s stroke and cognitive status at the time of nursing home admission. Data on residents who had stroke or developed cognitive impairment subsequent to admission were not collected as nursing home placement for such residents was not considered to be related to PSCI [30,31].

Secondly, the HIQA national standards for residential care settings require nursing homes to undertake a comprehensive assessment by an appropriately qualified healthcare professional of the health, personal and social care needs of a resident or a person who intends to be a resident immediately before or upon the person’s admission to the nursing home [32]. This includes a history of hospital and GP recorded diagnoses, including stroke, as well as a standardised assessment of cognitive function. HIQA national standards require that the tool to measure cognitive function is validated, reliable and internationally comparable. All nursing homes that participated in this survey used the Mini Mental State Examination (MMSE) to assess residents’ cognitive function as part of the comprehensive admission assessment of residents’ care needs [33]. The results of these comprehensive assessments are recorded in residents’ admission charts. By taking the results from the comprehensive assessments undertaken by a qualified professional using a tool to measure cognitive function that is validated and reliable [34,35,36], we felt confident that we obtained a reliable estimate of stroke and cognitive function at the time of nursing home admission. Given the survey’s focus on resident’s stroke and cognitive status at the time of nursing home admission, along with the availability of reliable estimates of stroke and cognitive impairment status at the time of admission from resident’s admission charts, individual assessments of residents were not required for the purpose of this study. Furthermore, previous Irish research in nursing homes found residents’ cognitive and functional impairments resulted in a 7% response rate with residents [20]. Finally, face–to–face interviews, as opposed to a telephone or a postal survey, were conducted for quality assurance purposes, to reduce the likelihood of incomplete data and to improve response rates [37,38,39].

The questionnaire distinguished between the number of residents with cognitive impairment that did not meet the criteria for dementia (cognitive impairment no dementia (CIND)) and the number of residents with dementia on admission. The distinction between CIND and dementia was based on the Diagnostic and Statistical Manual of Mental Disorders, Fifth Edition (DSM–5), criteria for mild or major neurocognitive disorder (see Additional File 1: Survey Questionnaire Table of definitions applied in the questionnaire). That is, whether an individual suffered either modest or severe cognitive decline in one or more cognitive domain as judged by a clinician with a validated measure, and if this decline was severe enough to interfere with independence in complex instrumental activities of daily living, such as paying bills and managing medications [40]. The MMSE assessment of cognition used by nursing homes participating in this study has a score range from 0 to 30 [33]. In conjunction with clinician oversight and interpretation, this tool has been shown to be an accurate screening measure of mild to moderate cognitive impairment and dementia [34,35,36]. There are a number of cut–offs proposed to assign scores into various categories of cognitive impairment. For the purposes of this study, we adopted the three group categorisation of MMSE scores recommended by Tombaugh and McIntyre [35] (<18 severe impairment; 18–23 mild–moderate impairment; 24–30 no impairment) along with the clinical judgement of the appropriately qualified healthcare professional as recorded in the resident’s admission chart. To support Directors of Nursing (DONs), the DSM–5 distinction between CIND and dementia was included in the questionnaire and referred to during the face–to–face interviews (see Additional File 1: Survey Questionnaire Table of definitions applied in the questionnaire). Stroke included ischaemic or haemorrhagic stroke. The questionnaire also distinguished between the number of residents with cognitive impairment with and without functional impairment (defined by impairment in Activities of Daily Living (ADLs) including bathing, dressing, toileting, transfers, continence and feeding.) [41]. To ensure the feasibility of the survey, during the development of the questionnaire both the questionnaire and data collection process were discussed with the Nursing Committee of the national representative body for the private and voluntary nursing home sector in Ireland, Nursing Homes Ireland (NHI).

### 2.4. Ethical Approval

This study was conducted in accordance with the Declaration of Helsinki. Ethical approval for this study was granted by the Research Ethics Committee of the Royal College of Surgeons in Ireland in March 2018 (reference number: REC 1477b). Written informed consent was obtained from all study participants.

### 2.5. Statistical Analysis

The analysis established prevalence estimates of cognitive impairment, as well as CIND and dementia estimates for residents with and without stroke on admission to nursing homes in Ireland. The corresponding 95% confidence intervals around these estimates were also estimated. Chi–squared tests were used to compare the differences in cognitive impairment prevalence estimates as well as CIND and dementia estimates among residents with and without stroke on admission. Statistical analysis was performed using Stata version 14.0. Two–sided significance at *p* < 0.05 was assumed.

## 3. Results

The age and sex distributions of residents are presented in Table 1. The majority of residents were female (62.5%; N = 402). Over 80% of residents in our sample were 75 years of age or older (N = 516), with those aged 85 years and older forming the biggest age group of residents (44.3%; N = 285). Just over 17% of residents experienced a stroke prior to admission to the nursing home (95% CI = 14.3–20.2%; N = 111). Of those residents with stroke, nearly a third (32.4%; 95% CI = 17.1%–47.7%; N = 36) had been admitted in the previous 12 months, and most of these residents (80.6%; 95% CI = 66.2–95.0%; N = 29) had been admitted directly from hospital. Functional impairment appeared to be quite high among those residents who experienced stroke prior to admission, with the majority of residents with stroke reported to have some level of impairment in activities of daily living, such as bathing, dressing, toileting, transfers, continence or feeding (87.4%; 95% CI = 81.2–93.6%; N = 97). Over 61% of all residents were reported to have cognitive impairments on admission (95% CI = 57.5–65.0%; N = 394). This comprised 52.7% of residents who were reported to have dementia on admission (95% CI = 48.9–56.6%; N = 339) and 8.6% of residents who were reported to have CIND (95% CI = 6.4–10.7%; N = 55) [40].

As presented in Table 2, of those residents who experienced stroke prior to admission, nearly 84% were reported to have a cognitive impairment on admission (83.8%; 95% CI = 76.9–90.6%; N = 93). In sharp contrast, among residents that had not experienced stroke, 56.6% were reported to have cognitive impairment on admission; this represented a significantly lower proportion (95% CI = 52.4–60.8%; N = 301; X^2^ (1) = 28.64; *p* < 0.001).

The proportions of residents reported to have dementia and CIND were also found to be significantly higher among those who experienced stroke when compared to those who did not experience stroke prior to admission. For example, of those residents that had experienced stroke prior to admission, 66.7% were reported to have dementia (95% CI = 57.9–75.4%; N = 74). In contrast, 49.8% of those who had not experienced stroke prior to admission were reported to have dementia (95% CI = 45.6–54.1%; N = 265; X^2^ (1) = 10.47; *p* < 0.01). In tandem with this, 17.1% of residents that had experienced stroke were reported to have CIND (95% CI = 10.1–24.1%; N = 19), whereas 6.8% of residents that had not experienced stroke prior to admission were reported to have CIND (95% CI = 4.6–8.9%; N = 36; X^2^ (1) = 12.58; *p* < 0.01). Finally, of those residents with stroke and cognitive impairment, the survey found that nearly 85% were reported to also have functional impairment (84.9%; 95% CI = 77.7–92.2%; N = 79).

## 4. Discussion

This study estimates the prevalence of cognitive impairment and dementia upon admission to nursing home for residents with and without stroke. The findings suggest that, in Ireland, nearly one in five nursing home residents have experienced stroke prior to admission. Given that the national and international estimates of the prevalence of stroke among nursing home residents include stroke cases both before and after nursing home admission, the estimate of 17% (95% CI = 14.3–20.2%; N = 111) found in this study appears to be consistent with that found in the international literature (18–25%) [20,21,42,43,44].

A key finding from the study is that the prevalence of cognitive impairment among Irish nursing home residents that experienced stroke appears to be significantly higher than that among those residents that had not experienced stroke prior to admission. It would appear that over 8 in 10 nursing home residents that experienced stroke prior to admission have some degree of cognitive impairment. When the results were broken down by the extent of cognitive impairment, the findings were similar; that is, the prevalence of both CIND and dementia was significantly higher among residents that experienced stroke when compared to those who had not experienced stroke prior to nursing home admission.

Examining the overall nursing home population, it appears that the prevalence of cognitive impairment in the nursing home population found in this study (61%; 95% CI = 57.5–65.0%; N = 394) is consistent with findings from the international literature employing similar methods. Studies that derive estimates from chart reviews and interviews with staff suggest that the prevalence of cognitive impairment and dementia ranges between 53% and 67% of the nursing home population [45,46,47,48,49,50]. Unlike this study, these international studies have not examined the prevalence of cognitive impairment in the stroke–specific population compared with those in nursing homes who have not had a stroke prior to admission.

Recent analysis of the long–term trends in the characteristics of stroke survivors discharged to nursing home care has found that the proportions of stroke survivors with functional disability moving to nursing home care has been consistently high (93–90%) over a period of more than 20 years (1995–2008) [17]. However, this analysis did not examine the extent of cognitive impairment amongst stroke survivors, nor the overlap between cognitive and functional impairments among stroke survivors in nursing homes. Our findings indicate that the majority of nursing home residents with stroke and cognitive impairment also have functional impairment, suggesting stroke survivors residing in Irish nursing homes may be particularly dependent and have considerable care needs, and therefore require resource–intensive service provision to meet these care needs.

### Strengths and Limitations

A major strength of this study is the use of a nationally representative sample to estimate the prevalence of cognitive impairment post–stroke on admission to nursing homes in Ireland. The study sample reflected both the age and sex distribution of the national nursing home population. Department of Health statistics suggest that, in Ireland, 65% of all nursing home beds are occupied by females and 70% of residents are aged 80 years and over [51]. In our study sample, 62.5% of residents were female and over 80% of residents were 75 years of age or older, with those aged 85 years and older forming the biggest age group of residents. The prevalence of cognitive impairment post–stroke upon admission to nursing homes is an area in which there is a dearth of research, yet it may have considerable implications for health service planners, as the demand for nursing home placement for those with PSCI is likely to increase in the coming decades in line with population ageing [22,23,24].

The survey questionnaire used in this study collected aggregated data on nursing home residents. Individual–level data were not collected. Therefore, it was not possible to undertake multivariable regression analysis to examine the extent to which the prevalence rates estimated vary by residents’ age, stroke severity or other socio–demographic factors. Furthermore, cognitive impairment was not measured by the individual objective neuropsychological assessment of residents for the purposes of the study. Data on cognitive impairment were taken from the results of MMSE administration, undertaken as part of the resident’s comprehensive assessment by a qualified professional. Data on stroke were also taken from the results of the comprehensive assessment. The results of the comprehensive assessment were recorded in residents’ admission charts and aggregated by Directors of Nursing. Consequently, the findings may under–estimate the prevalence of cognitive impairment and stroke at the time of admission. However, individual neuropsychological assessments would have placed an unnecessary burden on residents, as such assessments were not required for the purpose of this study. As the survey was conducted to establish a prevalence estimate of cognitive impairment among residents with and without stroke upon admission to nursing home, it was important that the survey collected data on residents’ stroke and cognitive status at the time of nursing home admission, not subsequent to admission. Had individual neuropsychological assessments been undertaken for the purposes of this study, this would have produced an estimate of the current prevalence of cognitive impairment amongst residents with and without stroke, opposed to an estimate of the prevalence of cognitive impairment among residents with and without stroke upon admission to nursing home. Furthermore, the study findings are in line with estimates from previous national and international studies, which employed similar methods to derive separate estimates of the prevalence of stroke and of cognitive impairment among nursing home residents.

The survey findings suggest a need for further research in this area in order to inform and support policy and practice in the nursing home sector. For example, as the survey found the prevalence rates of cognitive impairment and dementia to be significantly higher among residents that have experienced stroke compared with residents that have not experienced stroke, this suggests a need to investigate further the specific care needs of residents with stroke and cognitive impairment and how these can be met in the nursing home setting. Furthermore, while it was not possible in this study, it would be beneficial to examine how the prevalence rates of cognitive impairment and dementia among stroke survivors residing in nursing homes vary depending on such aspects as age, first or recurrent stroke, and stroke severity. This could support the development of specific interventions for a cohort of residents who are likely to have higher rates of cognitive impairment and dementia post–stroke, and consequently present with considerable care needs during nursing home placement.

## 5. Conclusions

The findings of this study suggest that the majority of nursing home residents that experienced stroke prior to admission have cognitive impairment. The prevalence of cognitive impairment among nursing home residents who experienced stroke prior to admission appears to be significantly higher than among those residents who had not experienced stroke prior to admission. The majority of residents with stroke and cognitive impairment were also reported to have functional impairment, suggesting considerable care needs for such residents. Since the prevalence of stroke, and stroke–related cognitive impairment and dementia, is likely to increase in line with population ageing in the coming decades, the study findings highlight an urgent need for health service planning for the potential increased demand for nursing home care, so as to meet the care needs of these stroke survivors. Finally, as there appears to be a dearth of literature in this area, further research on the characteristics and care needs of stroke survivors with cognitive impairment and dementia residing in nursing homes would help inform policy and practice in this area.

## Figures and Tables

**Table 1:** Sample description.

**Age and Sex Distribution of Residents % (N)**			
**Age Groups**	**Male**	**Female**	**Total**
<55 years of age	4.6 (11)	1.7 (7)	2.8 (18)
55–64 years of age	7.9 (19)	1.2 (5)	3.7 (24)
65–74 years of age	21.6 (52)	8.2 (33)	13.2 (85)
75–84 years of age	35.3 (85)	36.3 (146)	35.9 (231)
85+ years of age	30.7 (74)	52.5 (211)	44.3 (285)
Total	37.5 (241)	62.5 (402)	100.0 (643)
**Experienced stroke prior to admission**			
	**% Aged < 75 (95% CIs; N)**	**% Aged 75 + (95% CIs; N)**	**Total % (95% CIs; N)**
	3.9 (2.4–5.4; 25)	13.4 (10.7–16.0; 86)	17.3 (14.3–20.2; 111)
**Prevalence of stroke and functional impairment on admission**			
	**% Aged < 75 (95% CIs; N)**	**% Aged 75 + (95% CIs; N)**	**Total % (95% CIs; N)**
	18.0 (10.9–25.2; 20)	69.4 (60.8–77.9; 77)	87.4 (81.2–93.6; 97)

CIs: Confidence Intervals.

**Table 2:** Prevalence of stroke and cognitive impairment on admission to nursing homes in Ireland.

	Stroke % (95% CIs; N)	No Stroke % (95% CIs; N)	Total % (95% CIs; N)
CIND *	17.1 (10.1–24.1; 19)	6.8 (4.6–8.9; 36)	8.6 (6.4–10.7; 55)
Dementia	66.7 (57.9–75.4; 74)	49.8 (45.6–54.1; 265)	52.7 (48.9–56.6; 339)
**Cognitive Impairment Total**	**83.8 (76.9–90.6; 93)**	**56.6 (52.4–60.8; 301)**	**61.3 (57.5–65.0; 394)**

* CIND: Cognitive impairment no dementia. CIs: Confidence Intervals. The bold is show this is a total figure.

## Data Availability

The dataset used and analysed during the current study is available from the corresponding author on reasonable request.

## Supplementary Materials

The following are available online at https://www.mdpi.com/1660-4601/17/19/7203/s1, Additional File 1 Survey Questionnaire.

## Abbreviations

ADL	Activities of Daily Living
CIND	Cognitive Impairment No Dementia
DON	Directors of Nursing
DSM–5	Diagnostic and Statistical Manual of Mental Disorders, Fifth Edition
HIQA	Health Information and Quality Authority
HSE	Health Service Executive
NHI	Nursing Homes Ireland
PSCI	Post–Stroke Cognitive Impairment
