# Supplementary material for: The Prevalence of Cognitive Impairment on Admission to Nursing Home among Residents with and without Stroke: A Cross–Sectional Survey of Nursing Homes in Ireland
Source: Int J Environ Res Public Health. 2020 Oct 1;17(19):7203. doi: 10.3390/ijerph17197203 (PMC7579486; doi:10.3390/ijerph17197203)
Supplement: Supplementary file 1 [file ijerph-17-07203-s001.pdf]

The StrokeCog Study
National Survey of Nursing Homes

What is this survey?

This survey is being conducted by the Economic and Social Research Institute (ESRI) and Royal College of Surgeons in Ireland (RCSI). It is funded by the HRB and aims to identify the number of residents in nursing homes who have cognitive impairment and stroke (see table of definitions at the end of this questionnaire). We are interested in ischaemic or haemorrhagic stroke (we are excluding transient ischaemic attacks/mini strokes). We would like to invite Persons in Charge/Directors of Nursing to participate.

What is involved?

The questions do not look for information on individuals, but for aggregated data (e.g. total number of residents in certain categories). It will not be possible to identify any nursing home or any person in any publication arising from the survey.

Why is the survey important?

The information provided in the survey is critical as it will be used to estimate the costs of post-stroke cognitive impairment and dementia in nursing homes in Ireland. Without your participation we will not know the extent of stroke survivorship and cognitive impairment (including vascular dementia) amongst residents of nursing homes in Ireland. Consequently, without your participation it will not be possible to estimate the cost of post-stroke cognitive impairment and dementia in nursing homes in Ireland. As the population is ageing this information is important in helping healthcare planners and policy-makers plan for the future.

Thank you very much for taking the time to participate in this survey

1. Organisation Classification. Please tick one box

A. A Nursing Home as per Health (Nursing Homes) Act 1990 ☐
OR A Nursing Home as per Health Act 2004 Section 39 arrangement

B. The Health Service Executive ***OR*** A Nursing Home as per Health Act 2004 Section 38 arrangement ☐

3. Total number of beds in your nursing home:__________

2. Number of residents by age and sex

**Age**	**Male**	**Female**
Less than 55 years		
55 - 64 years		
65 - 74 years		
75 -84 years		
85 years and older		
**Total***		

***Total should not exceed bed total*

4. What is the weekly cost of a Fair Deal bed in your nursing home? €___________(Per week)

5. Number of residents that had a stroke prior to admission to nursing home

A. Total number of residents with Ischaemic stroke prior to admission to nursing home ________________

B. Total number of residents with Haemorrhagic stroke prior to admission to nursing home ________________

C. Total number of residents with unknown type of stroke prior to admission to nursing home ________________

Total number of residents that had a stroke prior to admission to nursing home ________________

These questions look at the number of residents with stroke prior to admission			
6. Of the residents with stroke prior to admission, what number had functional disabilities (impairment in Activities of Daily Living (ADLs)) and no cognitive issues?	Total:	Total aged <75:	Total aged 75+:
7. Of the residents with stroke prior to admission, what number had cognitive impairment no dementia?	Total:	Total aged <75:	Total aged 75+:
7b. Of these residents, what number had functional disabilities?	Total:	Total aged <75:	Total aged 75+:
8. Of the residents with stroke prior to admission, what number had a Dementia?	Total:	Total aged <75:	Total aged 75+:
8b. Of these residents, what number had functional disabilities?	Total:	Total aged <75:	Total aged 75+:
9. What number of residents had cognitive impairment no dementia without evidence of stroke prior to admission?	Total:	Total aged <75:	Total aged 75+:
10. What number of residents had dementia without evidence of stroke prior to admission?	Total:	Total aged <75:	Total aged 75+:

These questions look at source of admission for residents admitted in last 12 months			
	Community (Resident's home):	Acute hospital:	Long stay hospital/ nursing home:
11. What was the source of admission for residents with stroke, functional disabilities and no cognitive issues?			
12. What was the source of admission for residents with stroke and cognitive impairment no dementia?			
12b. Of these residents, what number had functional disabilities?			
13. What was the source of admission for residents with stroke and dementia?			
13b. Of these residents, what number had functional disabilities?			

These questions look at the number residents who died in the last 12 months	
	Number:
14. What number of residents with stroke, functional disabilities and no cognitive issues died in the last 12 months?	
15. What number of residents with stroke and cognitive impairment no dementia died in the last 12 months?	
15b. Of these residents, what number had functional disabilities?	
16. What number of residents with stroke and dementia died in the last 12 months?	
16b. Of these residents, what number had functional disabilities?	

These questions look at discharge destination for residents discharged in the last 12 months (excluding those who died)			
	Community (Resident's home):	Acute hospital:	Long stay hospital/ nursing home:
17. What was the discharge destination for residents with stroke, functional disabilities and no cognitive issues?			
18. What was the discharge destination for residents with stroke and cognitive impairment no dementia?			
18b. Of these residents, what number had functional disabilities?			
19. What was the discharge destination for residents with stroke and dementia?			
19b. Of these residents, what number had functional disabilities?			

These questions look at length of stay for residents discharged or died in the last 12 months				
	< 3 months	3 - <12 months	12 months - <4 years	4 or more years
20. What was the length of stay for residents with stroke, functional disabilities and no cognitive issues?				
21. What was the length of stay for residents with stroke and cognitive impairment no dementia?				
21b. Of these residents, what number had functional disabilities?				
22. What was the length of stay for residents with stroke and dementia?				
22b. Of these residents, what number had functional disabilities?				

Thank you for taking the time to complete this survey

Table of definitions applied in questionnaire	
**Cognitive impairment no dementia (CIND) and Dementia.**	
The questionnaire applies the DSM-5 criteria[1] for cognitive impairment no dementia and Dementia.	
**Cognitive impairment no dementia (CIND)**	Evidence of **modest cognitive decline** in one or more cognitive domains (see domains below) based on: 1. Concern of the individual, a knowledgeable informant, or the clinician that there has been a mild decline in cognitive function; and 2. A modest impairment in cognitive performance, documented by a standardized neuropsychological testing or, in its absence, another quantified clinical assessment.
	**Cognitive deficits are not severe enough to interfere with functional independence in everyday activities**. That is, individuals with CIND will have preserved independence, although there may be subtle interference with function or a report that tasks require more effort or take more time than previously. **Complex instrumental activities of daily living such as paying bills or managing medications are preserved**, but greater effort, compensatory strategies or accommodation may be required to maintain independence.
	The cognitive deficits do not occur exclusively in the context of a delirium.
	The cognitive deficits are not better explained by another mental disorder (e.g. major depressive disorder, schizophrenia).
**Dementia**	Evidence of **significant cognitive decline** in one or more cognitive domains (see domains below) based on: **1.** Concern of the individual, a knowledgeable informant, or the clinician that there has been a significant decline in cognitive function; **and 2.** A substantial impairment in cognitive performance, documented by a standardized neuropsychological testing or, in its absence, another quantified clinical assessment.
	**Cognitive deficits are sufficient enough to interfere with independence in everyday activities** such that others will have to take over tasks that the individuals were previously able to complete on their own (at a minimum requiring assistance with complex instrumental activities of daily living, such as paying bills or managing medications).
	The cognitive deficits do not occur exclusively in the context of a delirium.
	The cognitive deficits are not better explained by another mental disorder (e.g. major depressive disorder, schizophrenia).
**Cognitive domains**	
**Complex attention and processing speed**	Sustained attention, divided attention, selective attention, information processing speed.
**Frontal-executive function**	Planning, decision-making, working memory, responding to feedback/error correction, novel situations, over-riding habits, mental flexibility, judgement.
**Learning and memory**	Immediate memory, recent memory (including free recall, cued recall) and recognition memory.
**Language**	Naming, expressive, grammar and syntax, receptive language.
**Visuo-constructional-perceptual ability**	Construction, visual perception and reasoning.
**Praxis-gnosis-body schema**	Praxis, gnosis, right/left orientation, calculation ability, body schema, facial recognition.
**Social cognition**	Recognition of emotional and social cues, appropriate social inhibitions, theory of mind, empathy.
**Stroke**	
For this questionnaire we are interested in Ischaemic or Haemorrhagic stroke. We are excluding transient ischaemic attacks/mini-strokes.	
**Functional disabilities**	
Impairment in Activities of Daily Living (ADLS). These ADLs include bathing, dressing, toileting, transfers, continence and feeding.	

[1] *American Psychiatric Association. Diagnostic and Statistical Manual of Mental Disorders, Fifth Edition: DSM-5. Washington, CD: American Psychiatric Publishing, 2013.*
